# Bacterial Bioluminescence: Light Emission in *Photobacterium phosphoreum* Is Not Under Quorum-Sensing Control

**DOI:** 10.3389/fmicb.2019.00365

**Published:** 2019-03-04

**Authors:** Lisa Tanet, Christian Tamburini, Chloé Baumas, Marc Garel, Gwénola Simon, Laurie Casalot

**Affiliations:** Aix Marseille Univ., Université de Toulon, CNRS, IRD, MIO UM 110, Marseille, France

**Keywords:** bacterial bioluminescence, quorum sensing, *Photobacterium phosphoreum*, *lux* genes, high pressure

## Abstract

Bacterial-bioluminescence regulation is often associated with quorum sensing. Indeed, many studies have been made on this subject and indicate that the expression of the light-emission-involved genes is density dependent. However, most of these studies have concerned two model species, *Aliivibrio fischeri* and *Vibrio campbellii*. Very few works have been done on bioluminescence regulation for the other bacterial genera. Yet, according to the large variety of habitats of luminous marine bacteria, it would not be surprising to find different light-regulation systems. In this study, we used *Photobacterium phosphoreum* ANT-2200, a piezophilic bioluminescent strain isolated from Mediterranean deep-sea waters (2200-m depth). To answer the question of whether or not the bioluminescence of *P. phosphoreum* ANT-2200 is under quorum-sensing control, we focused on the correlation between growth and light emission through physiological, genomic and, transcriptomic approaches. Unlike *A. fischeri* and *V. campbellii*, the light of *P. phosphoreum* ANT-2200 immediately increases from its initial level. Interestingly, the emitted light increases at much higher rate at the low cell density than it does for higher cell-density values. The expression level of the light-emission-involved genes stays constant all along the exponential growth phase. We also showed that, even when more light is produced, when the strain is cultivated at high hydrostatic pressure, no change in the transcription level of these genes can be detected. Through different experiments and approaches, our results clearly indicate that, under the tested conditions, the genes, directly involved in the bioluminescence in *P. phosphoreum* ANT-2200, are not controlled at a transcriptomic level. Quite obviously, these results demonstrate that the light emission of the strain is not density dependent, which means not under quorum-sensing control. Through this study, we point out that bacterial-bioluminescence regulation should not, from now on, be always linked with the quorum-sensing control.

## Introduction

**FIGURE 1:**
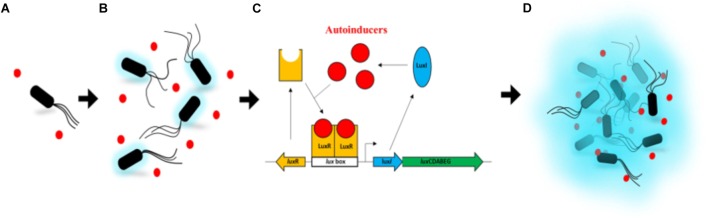
Activation of the *lux* operon by LuxR and LuxI in *Aliivibrio fischeri*. **(A)** At low cell density, the autoinducers (3OC6-HSL – red dots), produced by LuxI, diffuse through the cell membrane into the growth medium. **(B)** As the cell growth continues, the autoinducers in the medium start to accumulate in a confined environment. A very low intensity of light can be detected. **(C)** When enough autoinducers have accumulated in the medium, they can re-enter the cell where they directly bind the LuxR protein to activate *lux*ICDABEG expression. **(D)** High levels of autoinducers activate the luminescent system of *A. fischeri*. A high intensity of light can be detected. Based on Li and Nair (2012) (authorization of use license number 4454260086749).

Quorum sensing (in short designed as QS) is the regulation of gene expression in response to fluctuations in cell-population density as defined by Miller and Bassler (2001). The QS is often described as a way to communicate for bacteria. Actually, it is more the capability of the bacterial population to synchronize an individual behavior using small hormone-like chemical molecules called autoinducers. QS has been discovered by studying bacterial bioluminescence even if it was not, by then, defined as QS yet (Kempner and Hanson, 1968; Nealson et al., 1970). Indeed, using cultures, freshly inoculated with the bioluminescent bacterium *Aliivibrio fischeri* (previously identified as *Vibrio* or *Photobacterium fischeri*), Kempner and Hanson (1968) observed that the exponential growth phase starts straight on, i.e., without any latency phase. On the contrary, the light emission does not evolve until half of the logarithmic phase where it, then, increases quickly. This phenomenon of shift, between growth and luminescence, has already been reported 20 years earlier (Farghaly, 1950), but the nature of this uncoupling was not hypothesized at that time. It was later shown that, in fact, the luminescence was initiated by the accumulation of the autoinducer, which is synthesized by the bacteria and excreted into the medium (Nealson et al., 1970; Rosson and Nealson, 1981). It results that the light seems to be emitted only from a certain cell density allowing an important amount of these autoinducers to be produced. Since these studies, the whole genetic mechanism for *A. fischeri* has been well described as summarized thereafter. The LuxI-LuxR QS system directly regulates the expression of the *lux* operon, required for the light production in *A. fischeri* (Figure 1). LuxI, the autoinducer synthase, produces the *N*-(3-oxohexanoyl) homoserine lactone (3OC6-HSL), which belongs to the acyl-homoserine-lactone (AHL) family (Eberhard et al., 1981; Engebrecht and Silverman, 1984). LuxR is the cytoplasmic autoinducer receptor/DNA binding transcriptional activator (Engebrecht et al., 1983). When the 3OC6-HSL reaches a critical, threshold concentration, it binds to LuxR and this complex activates the transcription of the *lux* operon (Stevens and Greenberg, 1997). Two additional QS systems, AinS-AinR and LuxS-LuxP/Q, indirectly control luminescence by modulating *lux*R transcription (Verma and Miyashiro, 2013). Firstly referenced to as “autoinduction” (Nealson et al., 1970), the term quorum sensing was coined later by Fuqua et al. (1994). It is now common knowledge among microbiologists that QS systems, analogous to the one described above, regulate gene expression in a great variety of gram-negative Bacteria (Hastings and Greenberg, 1999), as well as in gram-positive Bacteria (Miller and Bassler, 2001) and more recently in Archaea (Zhang et al., 2012). QS is also known to regulate the bacterial-pathogen behavior, including, for instance, virulence-gene expression, biofilm formation, swarming, antibiotic production, and antibiotic resistance. Recently, there have been extraordinary advances in our understanding of the genetic, genomic, biochemistry, and signal diversity of QS (Whiteley et al., 2017). For more details, see reviews about QS (Whitehead et al., 2001; Waters and Bassler, 2005; Li and Nair, 2012; Rajput et al., 2015).

Despite the long-standing interest in bioluminescent bacteria and the regulation of their light, the vast majority of the studies have concerned only two model organisms: *A. fischeri* and *Vibrio campbellii* (previously identified as *Beneckea* or *Vibrio harveyi*). However, luminous bacteria cannot be confined to these two species. Currently, about 25 species of luminous bacteria have been discovered across five genera in three families of the Gammaproteobacteria: Shewanellaceae (*Shewanella*), Enterobacteriaceae (*Photorhabdus*), and Vibrionaceae (*Aliivibrio*, *Photobacterium*, and *Vibrio*) (Dunlap and Urbanczyk, 2013). In marine environments, luminous bacteria are widely distributed. They can be isolated from seawater, sediments or suspended, and sinking particles, but bioluminescent bacteria are also well known as symbionts with animals (Nealson and Hastings, 1979; Andrews et al., 1984; Dunlap and Urbanczyk, 2013). Quite obviously, according to the large variety of habitats, it would not be surprising to find, in other luminous bacteria, light-regulation systems different than the two most studied model organisms, *A. fischeri* and *V. campbellii*. Due to its historic discovery with luminous bacteria, QS control is too often associated to all bioluminescent bacteria. Yet, some previous works on *Photobacterium* have suggested different kind of regulation (Katznelson and Ulitzur, 1977; Rosson and Nealson, 1981). To date, only rare studies have dealt with the bacterial-light regulation in *Photobacterium* species, as for instance the work of Dunn et al. (2015) on *Photobacterium leiognathi*.

In this study, we have chosen to use *Photobacterium phosphoreum* ANT-2200, a luminous piezomesophilic bacterium (Martini et al., 2013), which entire genome has been sequenced (Zhang et al., 2014). We focused on the relationship between growth and light emission through physiological, genomic and, transcriptomic approaches at atmospheric pressure. Since its growth and light emission have been well described previously at high hydrostatic pressure (Martini et al., 2013), we also tested *lux* genes expression at this condition.

## Materials and Methods

### Bacterial Strain and Culture Conditions at Atmospheric Pressure

*Photobacterium phosphoreum* ANT-2200 (16S rDNA GenBank accession number EU881910) was isolated from seawater collected in the Northwestern Mediterranean Sea at the ANTARES neutrino telescope site (42°54′N/06°06′E) at 2200-m depth (13°C). See Al Ali et al. (2010) for details. For the first experiment, conducted at atmospheric pressure, the strain was cultured in a mineral-salt medium (ONR7a modified), close to the environmental composition and requiring the preparation of three different solutions as described hereafter. Solution 1: 22.79 g NaCl, 3.98 g Na2SO4, 0.72 g KCl, 0.8 g NH4Cl, 0.2 g Na2HPO4.2H2O, 83 mg NaBr, 2.6 mg NaF, 31 mg NaHCO3, 27 mg H3BO3, 2 mL glycerol, and 10 mL Balch oligo-element (Balch et al., 1979) in 700 mL of distilled water (pH 7.5). Solution 2: 11 g MgCl2.6H2O, 1.46 g CaCl2.2H2O, and 24 mg SrCl2.6H2O in 300 mL of distilled water. Solution 3: 0.025 g FeSO4.7H2O in 10 mL of distilled water. The three solutions were autoclaved separately for 20 min at 121°C. Solution 1, solution 2 and 1 mL of the solution 3 were mixed after autoclave and 20 mL of Balch vitamins were added (Balch et al., 1979). Cultures were grown in 100-mL flasks containing 50 mL ONR7a modified medium, in a 19°C-temperature-controlled room with orbital shaking at 150 rpm.

### Growth and Bioluminescence Measurements

Bacterial growth was estimated by measuring the optical density at 600 nm in a Milton Roy Spectronic 401 spectrophotometer. The light emission was measured in a 96-well plate, with 150 μL of culture, in a luminometer Luminoskan Ascent (Lab Systems, Ascent Software Version 2.6), with the following parameters: PMT sensibility: 800 V, shaking: 10 s, reading time: 1 s. The light intensity is evaluated in relative light unit (RLU). Samples were taken regularly all along the growth. Each filtered volume was adjusted depending on the increase of the cell density. For samples 1, 2, 3, 4, and 5, 200, 100, 35, 20, and 7 mL were filtered on 0.2 μm cellulose-nitrate filters (Whatman), respectively. The experiment was done in duplicate. Less than 1% and less than 5% difference between both experiments were measured for the OD and the bioluminescence, respectively.

### High-Hydrostatic Pressure Experiments

For these experiments, due to the specificities of culturing under high-pressure conditions, and in order to reproduce the exact same experiment than Martini et al. (2013), a medium, richer than the ONR7a, was used. Procedures for culturing were performed as described by Martini et al. (2013). Briefly, cultures were incubated at 0.1 and 22 MPa of pressure and at 13°C. For the 22-MPa condition, cultures were placed into high-pressure bottles (HPBs). Each HPB contains three identical 5-mL plastic syringes and one 145-mL glass flask equipped with a rubber-septum cap to transmit the pressure. Each of the items contained a mix of 3/4 culture and 1/4 oxygen-saturated Fluorinert^TM^ FC-72 (3 M). Fluorinert^TM^ FC-72 was used as the oxygen supplier to ensure the growth and the luminescence of the bacterial strain in closed conditions (see Martini et al., 2013). The syringes were used for growth estimation by measuring the optical density (OD_600 nm_) and the flask was used for the RT-qPCR experiments. In order to avoid decompression-recompression of the samples, each HPB corresponded to one incubation time. Three samples were chosen as described in results. Each filtered volume was adjusted depending on the increase of the cell density. For samples T1, T2, and T3, 50, 35, and 25 mL were filtered on 0.2 μm cellulose-nitrate filters (Whatman), respectively.

### Quantitative RT-PCR

RNA extraction was performed with the High Pure RNA Isolation Kit (Roche Applied Science, United States) according to the supplier’s recommendations. The extracted RNAs were quantified at 260 nm using the BioSpec-Nano spectrophotometer (Shimadzu). The cDNAs were synthesized from the total extracted RNAs using random primers and the GoScript Reverse Transcription System (Promega) according to the supplier’s recommendations. For each sample, the amount of RNAs, added to the reaction mixture, was adjusted so that the amount of RNAs was identical for each sample. Thus, despite the difference in sampled volumes and cell density, the same quantity of RNAs from the five samples was used for the reverse transcription. The quantity of the various target cDNAs was quantified by qPCR (qPCR Master Mix, Promega) according to the supplier’s recommendations. Experiences were made using the CFX96 Bio-Rad and the associated software. Various plasmids were constructed by cloning the target genes in the pGEM-T-easy vector (Promega) according to the supplier’s recommendations (Supplementary Table S1). They were used for constituting the standard curves for qPCR allowing the quantification of the synthetized cDNAs. Each extracted RNA were analyzed through six technical replicates (three independent reverse transcriptions and two qPCR analyses per cDNAs).

## Results and Discussion

### Organization of Light-Emission-Involved Genes

**FIGURE 2:**
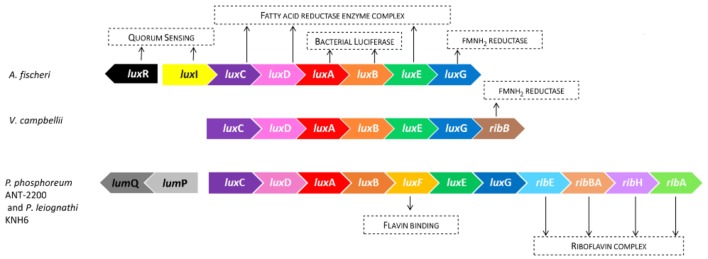
Comparison of the organization of the light-emission-involved genes in different luminous species. *A. fischeri* possesses the two regulatory genes (*lux*I/*lux*R), involved in the quorum sensing, in the vicinity of the *lux* operon. Whereas, at the same location, there are absent in *V. campbellii* and *Photobacterium* spp. *P. phosphoreum* ANT-2200, and *P. leiognathi* KNH6 organizations are identical.

Figure 2 represents the *lux* operon and its organization in the genome of three luminous marine bacteria: *A. fischeri*, *V. campbellii*, and *P. phosphoreum* ANT-2200. The genes *lux*CDABEG are common to all of them and code for the luciferase (AB) and for the fatty-acid-reductase polypeptides (CDEG) (Meighen, 1993). *Photobacterium* strains present some additional genes. For example, in *P. phosphoreum* ANT-2200, and in the majority of other bioluminescent *Photobacterium* species, the *lux*F gene is present between *lux*B and *lux*E (Mancini et al., 1988; Soly et al., 1988). Not found, to date, in any *Aliivibrio*, *Vibrio* or other bioluminescent bacteria, the *lux*F gene appears to be confined to the genus *Photobacterium* (Mancini et al., 1989; Ast and Dunlap, 2004). The *lux*F gene encodes a protein which is suggested to increase the emitted luminous intensity by binding to a bioluminescence inhibitor, the myrFMN (Bergner et al., 2015). Furthermore, *Photobacterium* species possess also the *rib* genes, involved in the riboflavin synthetase, which are located directly downstream *lux*G (Lee et al., 1994; Lin et al., 2001). The *rib* genes belong to the *lux* operon since they are co-transcribed from a promoter located upstream *lux*C (Sung and Lee, 2004). Such an organization is not surprising since riboflavin is the direct precursor of riboflavin 5′-mono-phosphate (FMN), the oxidized form of the substrate (FMNH_2_) used by bacteria in the bioluminescence reaction. This clearly underlines the importance of the *rib* genes and their links to the *lux* genes and the light reaction. The classic *rib*-gene organization in *Photobacterium* is *rib* E-B-H-A. However, it is worth noting that the absence of *rib*E is described for some *P. phosphoreum* strains (Urbanczyk et al., 2011; Dunlap, 2014) and not in some others (Lee et al., 1994; Sung and Lee, 2004; Zhang et al., 2014). Interestingly, in *V. campbellii*, only one gene, *rib*B, coding for a key step of the riboflavin-complex synthetase, can be found (Swartzman, 1990; Dunlap, 2014).

One of the main differences in the bioluminescence-gene organization concerns the regulatory genes. Some species, such as *A. fischeri*, present the regulatory genes, *lux*I and *lux*R, upstream the *lux*CDABEG cluster. In this case, the *lux*I gene is transcribed with *lux*C and therefore belongs to the *lux* operon. On the contrary, *lux*R is located further upstream the *lux* operon and is transcribed in the opposite direction. Both genes are involved in the quorum-sensing-regulation mechanism. Remarkably, in all the luminous *Photobacterium* species, *lux*I and *lux*R are absent upstream *lux*C (Dunlap, 2014). In *P. phosphoreum* ANT-2200, instead of these genes, other ones precede the *lux* operon: *lum*P, also called *lux*L, and *lum*Q. They form the lumazine operon, which runs in the opposite direction of the *lux* genes. The same organization was found in *P. leiognathi* (Lin et al., 1995). The importance of LumP in *P. phosphoreum* was established prior to its localization, and was shown to be involved in the wavelength, as well as in the intensity of the light emission (Gast and Lee, 1978; Prasher et al., 1990). On the contrary, the function of LumQ remains unknown (Dunlap, 2014), although it is thought to code for a DNA-binding protein (Lin et al., 1995). Nevertheless, the only absence of the regulatory genes in the vicinity of the *lux* operon does not mean an absence of QS. Indeed, as observed in *Photobacterium* species, both genes are absent in *V. campbellii* at this location. However, in this organism, a lag time before the increase of the light emission has been described (Greenberg et al., 1979). It also has been observed that a high concentration of autoinducers permits the activation of the *lux*-operon transcription. The involved mechanism is now well described and is quite different than *A. fischeri* one (Bassler et al., 1997; Whitehead et al., 2001; Defoirdt et al., 2008).

In view of these various examples, the only organization of the genes in *P. phosphoreum* ANT-2200 is not enough to confirm an independent-cell-density light control. In this goal, we designed a follow-up of the *luxrib*-gene expressions and bioluminescence kinetics throughout the growth.

### Growth and Bioluminescence of *P. phosphoreum* ANT-2200

**FIGURE 3:**
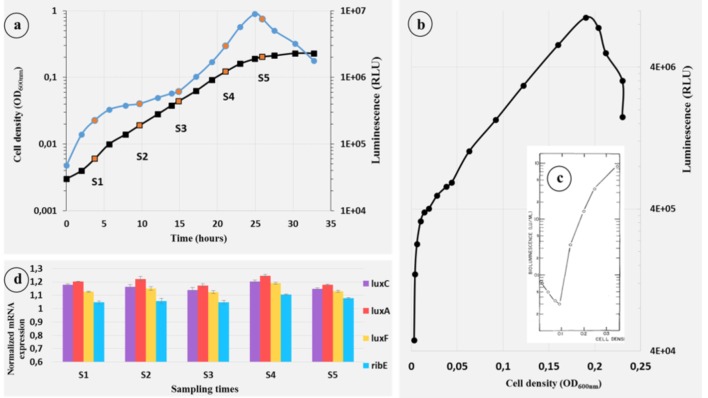
Growth, bioluminescence, and *luxrib*-gene-expression kinetics of *P. phosphoreum* ANT-2200 throughout the growth at atmospheric pressure. **(A)** Growth (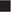 black) and bioluminescence (• blue) kinetics of *P. phosphoreum* ANT-2200 on ONR7a modified medium. Orange dots represent the sampling times S1 to S5. **(B)** Light emission of *P. phosphoreum* ANT-2200 according to the cell density. **(C)** Light emission of *A. fischeri* according to the cell density (Nealson, 1977). Authorization of use license number 4454241308645. **(D)** Expression level of the genes *lux*C, *lux*A, *lux*F, and *rib*E for sampling times S1–S5.

The growth and luminescence of *P. phosphoreum* ANT-2200 were measured over time in a mineral-salt medium (ONR7a) with glycerol as the only source of carbon and results are presented in Figure 3A. There is no lag time between the light emission and the growth as already observed for *P. leiognathi*. These results are in contrast with *A. fischeri* and *V. campbellii* results generally obtained, in which light emission appears when a certain quantity of cells is reached (Kempner and Hanson, 1968; Nealson et al., 1970; Katznelson and Ulitzur, 1977; Nealson, 1977; Engebrecht et al., 1983; Makemson, 1986; Meighen, 1999; Nackerdien et al., 2008). A light emission starting to increase at the same time than the growth means that the microorganism emits light even at really low cell density, which is not coherent with a density-dependent light control (Nealson, 1977).

In order to compare the light-emission kinetics of *P. phosphoreum* ANT-2200 with these of luminous bacteria under quorum sensing, we plotted the emitted light according to the cell density (Figure 3B), as Nealson (1977) did for *A. fischeri* (Figure 3C). For *P. phosphoreum* ANT-2200, the pattern completely contrasts to the one obtained for *A. fischeri*. Indeed, for the later, the light decreases from its initial level until the bacterial population reaches the required density – here 0.1 OD_600 nm_ – and then, considerably increases. On the contrary, for *P. phosphoreum* ANT-2200, the light, according to the cell density, strongly increases directly from its initial level. It is worth noting that, not only, it starts immediately, but also, it increases at a much higher rate at the low cell density – between OD_600 nm_ values of 0.003–0.01 – than it does for superior cell-density values – from 0.01 to 0.2. If it has been already suggested that *Photobacterium* spp. do not wait any quorum to emit light, this is, to our knowledge, the first report of higher specific luminescence for low rather than high cell density.

While the luminescence increases rapidly from the very beginning of the growth, we also noticed a slowdown period, from 5 to 14 h (Figure 3A), before increasing again to reach the maximum intensity at the end of the exponential growth phase after 25 h. Such slowdown period of light emission has already been described for *P. leiognathi* (Katznelson and Ulitzur, 1977; Dunn et al., 2015) when the cells were grown in rich-organic medium. Based on a previous work (Eberhard, 1972), they hypothesized the presence of an inhibitor contained into the rich-organic medium. In our study and using a minimal medium, the slowdown period of light emission could be explained by a molecule that accumulates in the medium of culture after a few hours before not being available anymore. Only bioluminescence would then be affected, since no slowdown phase is observed on the growth curve during this same period (Figure 3A). Amongst the potential molecules, one could imagine that myrFMN, by-product of the bioluminescence reaction, would compete with the substrate – as described by Wei et al. (2001) – before being scavenged by LuxF (Bergner et al., 2015), therefore allowing the light to increase again.

The phenotype of *P. phosphoreum* ANT-2200 presents so many differences with those of QS-controlled bioluminescent bacteria that it seems obvious to complete the follow-up of growth and light emission of *P. phosphoreum* ANT-2200 by a genetic approach.

### Quantitative RT-PCR on the Light-Emission-Involved Genes Throughout the Growth

A quantitative reverse-transcription PCR (RT-qPCR) was applied to monitor, all along the growth, the mRNA-expression level of the *luxrib* genes, the light-emission-involved genes in *P. phosphoreum* ANT-2200. Cells in different conditions, or under the influence of specific compounds, differ by their gene expression patterns and thus by their mRNA pools. One of the most important technique for the accurate quantification of gene expression is the fluorescent RT-qPCR (Muller et al., 2002). Indeed, RT-qPCR has become a routine and robust approach for measuring the expression of genes of interest (VanGuilder et al., 2008). In this objective, regular samples have been taken throughout the growth (orange dots in Figure 3A). Four genes were chosen for this experiment: *lux*C, the first gene of the operon, *lux*A, which codes for the α-subunit of the luciferase, *lux*F, specific to the *Photobacterium* genus and *rib*E, first gene of the *rib* cluster. In order to be sure to accurately compare the expression levels of each gene between the samples, they were normalized with the *rpo*D-gene expression. *rpo*D is an house-keeping gene and is often used to resolve phylogenetic analyses in *Photobacterium* clade (Ast et al., 2007; Bjornsdottir-Butler et al., 2016). The genes *lux*C, *lux*A, *lux*F, and *rib*E display a constant expression for every sample (Figure 3D) showing that they are not regulated during the growth in this condition. Furthermore, the first sample (S1) was performed at a very low optical density – 0.007 OD_600 nm_ – and yet, the mRNA amounts of all the targeted genes were high enough to be detected. Constant mRNA levels, even at low density, go against the hypothesis saying that a minimum cell density is required to activate the *lux*-operon transcription, which is coherent with the physiological observations.

Our results, obtained with *P. phosphoreum* ANT-2200, combined with recent studies with *P. leiognathi* (Dunn et al., 2015), confirm the absence of QS regulation for the bioluminescence in this genus. However, in some conditions, light intensity may vary. Therefore, despite the absence of regulation by the cell density, could the expression of the *luxrib* operon be regulated by other factors?

### RT-qPCR on the Light-Emission-Involved Genes at Atmospheric vs. High-Pressure Conditions

**FIGURE 4:**
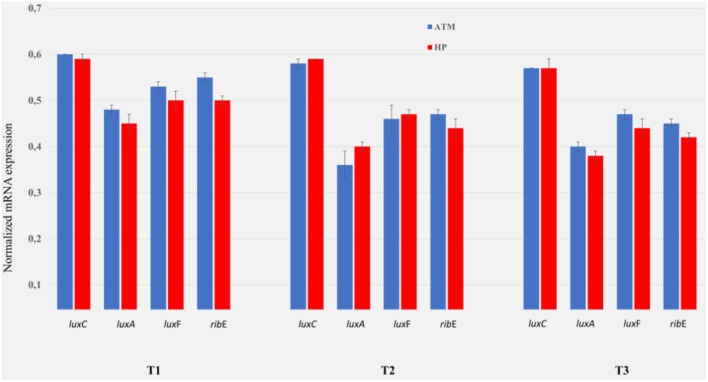
Comparison *luxrib*-gene-expression at atmospheric vs. high pressure of *P. phosphoreum* ANT-2200. Expression level of the genes *lux*C, *lux*A, *lux*F, and *rib*E at atmospheric (ATM, 0.1 MPa, blue) and at high hydrostatic pressure (HP, 22 MPa, red), for sampling times T1–T3. T1 corresponds to the initial time when the light emission is the same for both conditions (approximately 4 h). T2 and T3 correspond to the times when a bioluminescence-emission peak is reached at ATM (approximately 13 h) and HP (approximately 17 h) conditions, respectively. Reference bioluminescence and growth curves are available in Martini et al. (2013).

A previous study (see Figure 7 in Martini et al., 2013) showed that *P. phosphoreum* ANT-2200 produces three-times more light at 22 MPa – the *in situ* pressure condition – than at 0.1 MPa – the atmospheric pressure. The authors demonstrated that not only the bioluminescence was higher, under pressure, but also, that it occurs later on during the growth, comparatively to the results obtained at atmospheric pressure. In order to estimate if this variation can be related to a transcriptional regulation, we performed a RT-qPCR to compare the expression of the *luxrib* genes at 22 MPa vs. 0.1 MPa. Three sampling times have been chosen according to the light emission differences between the cultures. One corresponds to the initial time (T1) when the light emission is the same for both cultures. The two others correspond to the times when the bioluminescence-emission peak is reached at 0.1 and 22 MPa of pressure (T2 and T3). Despite a luminescence production three-times higher under 22 MPa, the expression levels of the genes *lux*C, *lux*A, *lux*F, and *rib*E do not show higher value than at 0.1 MPa (Figure 4). Therefore, the higher light production at 22 MPa cannot be explained by a transcriptional regulation of the *luxrib* genes. Other hypotheses could explain such a phenomenon. For example, the pressure role could still happen at a transcriptional level by regulating the expression of genes coding for proteins indirectly involved in the light emission. On the other hand, the pressure could also impact the structure and/or the function of proteins (Mozhaev et al., 1996), whether they are directly involved in the light emission or just associated to the quality of this light.

## Ecological Significance

Bioluminescence is considered to be a major factor in ecological interactions since 76% of the observed individuals in the water column have the capability of emitting light (Martini and Haddock, 2017). Knowledge of control mechanisms is important to the understanding of the selective advantage of luminescence and the ecology of the bacteria. With the discovering of the quorum sensing and the requirement of a minimum cell density to activate the bacterial light system, it was mentioned that free-living bacteria may have no ecological significance (Hastings and Greenberg, 1999). However, this hypothesis could be conceivable only if we considered that all bioluminescent bacteria are under quorum-sensing control.

Often described as the brightest species (Meighen, 1999; Cheng et al., 2010) and associated to midwaters and deep-sea habitats (Ruby and Morin, 1978; Urbanczyk et al., 2011), *P. phosphoreum* is found free-living or attached-to-particles in the seawater (Andrews et al., 1984) but never in a specialized light organ (Herring, 1982; Urbanczyk et al., 2011). As a matter of fact, *P. phosphoreum* ANT-2200 was found free in the deep waters of the Mediterranean Sea during a high level of bioluminescence (in relationship with a strong convection event) detected by the underwater neutrino telescope ANTARES (Tamburini et al., 2013). This result is coherent with the description of this species as the dominant bioluminescent microorganism in the Mediterranean Sea (Gentile et al., 2009). Moreover, the genus of *Photobacterium* has also been found as active even during a stable hydrological period suggesting an ecological benefit of bioluminescence (Martini et al., 2016). This finding agrees with one hypothesis on the ecological function of free-living bioluminescent bacteria: promoting their propagation and dispersal. According to this hypothesis, luminous bacteria growing on food particles visually mark their presence for higher trophic organisms, such as fish or zooplankton. These glowing particles have been shown to be preferentially ingested (Zarubin et al., 2012). The consumption of the particles by the predator would provide the bacteria a more suitable environment regarding the growth conditions and the nutrient accessibility (Nealson and Hastings, 1979). Therefore, attached bacteria are transferred to the nutritious guts of these macroorganisms, where they survive digestion and gain effective means for growth and dispersal. Thus, bioluminescence would be highly beneficial for marine bacteria, especially in food-deprived environments like the deep sea. This hypothesis can only be true if the light can be produced without QS control since, for free-living microorganisms, the conditions allowing the accumulation of the pheromone-like molecules can never be reached. To be able to produce light at very low concentration (therefore without QS control) will give *P. phosphoreum* this advantage.

## Conclusion

Through different experiments and approaches, our results clearly indicate that, under the tested conditions, the genes, directly involved in the bioluminescence in *P. phosphoreum* ANT-2200, are not controlled at a transcriptomic level. Quite obviously, this absence of regulation shows the absence of a cell-density control mechanism coherent with its free-living behavior. Because numerous studies about QS are linked to bioluminescence and due to its historic discovery with luminous bacteria, QS control is too often associated to all bioluminescent bacteria. We showed that this is clearly not the case with all light-emitting microorganisms, and that such automatic association should now be avoided.

## Data Availability

All datasets generated for this study are included in the manuscript and/or the Supplementary Files.

## Author Contributions

LT performed most of the experiments. CT and LC participated in the experimental design. CB and LC participated to experimental work on RT-qPCR. The high-pressure experiments were carried out by LT, MG, and CT. LT, GS, CT, and LC interpreted the results and wrote the manuscript. All authors reviewed the final version of the manuscript.

## Conflict of Interest Statement

The authors declare that the research was conducted in the absence of any commercial or financial relationships that could be construed as a potential conflict of interest.

## Supplementary Material

Table S1 Primer sequences and features of the target genes in *P. phosphoreum* ANT2200, used in RT-qPCR.
