# Supplementary material for: Bacterial Bioluminescence: Light Emission in Photobacterium phosphoreum Is Not Under Quorum-Sensing Control
Source: Front Microbiol. 2019 Mar 4;10:365. doi: 10.3389/fmicb.2019.00365 (PMC6409340; doi:10.3389/fmicb.2019.00365)
Supplement: Table S1 — Primer sequences and features of the target genes in P. phosphoreum ANT2200, used in RT-qPCR. [file Table_1.DOCX]

| **Target Genes** | **Primer Names** | **Primer sequences 5’ 🡪 3’** | **Fragment Sizes** | **PCR Hybridization Temperatures** |
| --- | --- | --- | --- | --- |
| *lux*C | luxC_586__F | CACCATCCAATTAGCCGTTC | 133 pb | 58 °C |
|  | luxC_718__R | TGATGCAATTAAATGGGCAA |  |  |
| *lux*A | luxA_187__F | CTTGGTCGAACCACCAAACT | 144 pb | 60 °C |
|  | luxA_330__R | GTGTTGTGCGTGGCTTGTAC |  |  |
| *lux*F | luxF_253__F | GCAGCGAAATATGCATTACC | 130 pb | 60 °C |
|  | luxF_382__R | AGATTTGGTTCGACACCAGC |  |  |
| *rib*E | ribE_344__F | CTGGGCGTGCGATAGATATT | 173 pb | 60 °C |
|  | ribE_516__R | TTGAAACGACAATCGCTGAC |  |  |
| *rpo*D  ANT2200 | rpoD_ANT2200_F | ACGCCGAAGTAAATGACCAC | 208 pb | 60 °C |
|  | rpoD_ANT2200_R | GTAGAAAGCGAGATCGGTCG |  |  |

**PCR program:** 1 min at 98 °C; 30 cycles of 30 sec at 98 °C, 30 sec at the corresponding hybridization temperature and, 20 sec at 72 °C; 5 min at 72 °C
